# 5' long terminal repeat (LTR)-selective methylation of latently infected HIV-1 provirus that is demethylated by reactivation signals

**DOI:** 10.1186/1742-4690-3-69

**Published:** 2006-10-12

**Authors:** Takaomi Ishida, Akiko Hamano, Tsukasa Koiwa, Toshiki Watanabe

**Affiliations:** 1 Laboratory of Tumor Cell Biology, Department of Medical Genome Sciences, Graduate School of Frontier Sciences, The University of Tokyo, 4-6-1 Shirokanedai, Minato-ku, Tokyo 108–8639, Japan

## Abstract

We previously described selective hypermethylation of the 5'-long terminal repeat (LTR) of HTLV-1 provirus *in vivo *and *in vitro*. This prompted us to analyze CpG methylation of the two LTRs of the HIV provirus in chronically infected cell lines. The results demonstrate selective hypermethylation of the 5' LTR of the HIV provirus in ACH-2 cells. Moreover, induction of viral gene expression by TNF-α resulted in demethylation of the 5'-LTR. These results suggest that selective epigenetic modification of the 5'LTR of the HIV-1 provirus may be an important mechanism by which proviral activity is suppressed.

## Findings

With the use of highly active anti-retroviral therapy (HAART) for HIV-infected individuals, greater control of viral replication is now possible. The widespread use of HAART has led to a substantial decline in the incidence of acquired immunodeficiency syndrome (AIDS) and AIDS-related mortality [1-6]. This development has led to considerable optimism [7], but complete eradication of HIV from an infected individual is difficult to achieve because there are latently infected resting CD4+ T cells carrying replication-competent HIV resistant to HAART [8-11]. A better understanding of mechanisms underlying latency and reactivation of HIV might yield information on how to overcome the resistance of latent HIV to treatment, and this would contribute to the goal of containment or purging of HIV.

Epigenetic control is thought to be involved in latent infection of HIV. Epigenetic mechanisms result in the heritable silencing of genes without a change in their coding sequence. Three systems, including DNA methylation, RNA-associated silencing and histone modification, are used to initiate and sustain epigenetic silencing [12]. Histone deacetylation is important for quiescence of HIV gene expression in infected resting CD4+ T lymphocytes. Blockade of histone deacetylase (HDAC) activity can stimulate the release of virus from latently infected CD4+ T-cells in vitro and, in combination with enfuvirtde, reduces the pool of CD4+ T-cells *in vivo*[13-15].

CpG methylation has been implicated in silencing of the integrated provirus genome [16,17] as well as in regulation of many imprinted genes [18]. Demethylation induced by an inhibitor of DNA methyltransferase, 5-Azacytidine (5-AzaC), was shown to reactivate a latent provirus [19]. *In vitro *studies have shown that DNA methylation suppresses the promoter activity of the HIV-1 long terminal repeat (LTR) [20-23], suggesting that CpG methylation may play an important role in viral latency *in vivo*.

Cytokines such as TNF-α induce HIV gene expression in chronically infected T cell lines [24,25], as well as in latently infected lymphocytes *in vivo *[26,27]. Using chronically infected T cell lines, we investigated CpG methylation of provirus LTR and its relationship to regulatory mechanisms that reactivate the latent HIV provirus. We found that CpG sites in the 5'-LTR are selectively hypermethylated, and that TNF-α-induced reactivation is associated with demethylation of the 5' LTR. Our observations provide clues to the mechanism of signal-mediated demethylation and reactivation of latent HIV.

**Figure 1:**
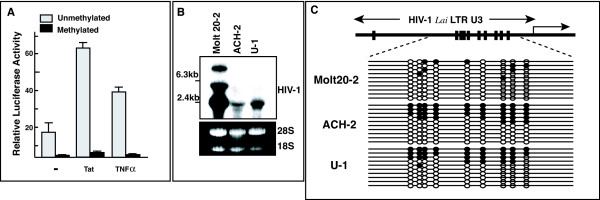
Promoter activity and CpG methylation of HIV LTR. **A**. Suppression of HIV LTR promoter activity by CpG methylation *in vitro*. HIV LTR-Luc plasmid, with or without *in vitro *methylation by SssI methylase, was transiently transfected into Jurkat cells with pRL-tk-Luc plasmid. Representative results of triplicate experiments are presented with standard deviation. Relative luciferase activity was determined by dividing the activity of firefly luciferase by that of renilla luciferase. Three independent experiments gave almost identical results. **B**. Northern blot analysis of HIV mRNA. Expression of HIV mRNA was detected using HIV LTR probe (upper panel). Molt20-2 is a gift from Prof. T Shiota (Osaka University) and derived form Molt4 infected by HIV Lai. Lower panel, photograph of ethidium bromide stained samples. **C**. Results of CpG methylation analysis of integrated HIV provirus LTRs of chronically infected cells lines. Single line represents the results of one plasmid clone analyzed. Upper panel, Schematic map of the CpG sites in the U3 region of HIV-1 IIIB LTR. Closed and open circles indicate methylated and unmethylated CpG sites, respectively.

To evaluate the effects of CpG methylation on the promoter activity of the HIV LTR, we first tested the effects of *in vitro *CpG methylation of HIV LTR-luciferase constructs on activity in transient transfection assays. When transfected into Jurkat cells, the HIV LTR-Luc plasmid showed significant basal levels of luciferase activities, whereas SssI methylase-treated HIV LTR-Luc plasmid showed 100-fold lower luciferase activities (Fig. 1A). We also found that CpG methylation suppressed the LTR's response to activating agents such as HIV Tat or TNF-α. The small responses we observed might be due to incomplete methylation of CpG during SssI methylase treatment, since bisulfite-sequencing analysis of the SssI-treated plasmid detected some unmethylated copies (data not shown). Thus, CpG methylation of the HIV LTR suppresses both basal promoter activity and responses to activating stimuli; this confirms results of earlier studies [20-23].

We next asked if LTRs of integrated proviruses were methylated. Using bisulfite genomic sequencing [28], we analyzed methylation of each CpG site in the U3 region of the HIV-1 LTR in chronically infected cell lines. Primers used for amplification of the modified sense strand are: LTR forward primer (F-3): 5'-TTTGTTATATTTTGTGAGTTTGTAT-3' (nucleotide position: 200 to 224, 9285 to 9309), reverse primer (R-1), 5'-CAAAAAACTCCCAAACTCAAATCTA-3' (nucleotide position: 496 to 472, 9581 to 9557). Amplified products were cloned by the TA method followed by sequence analysis using an automated sequencer (Amersham Bioscience, Gene Rapid). The results showed various levels of CpG methylation in Molt 20-2, ACH-2 and U-1 cell lines, which correlated inversely with basal levels of viral gene expression (Fig. 1B and C).

**Figure 2:**
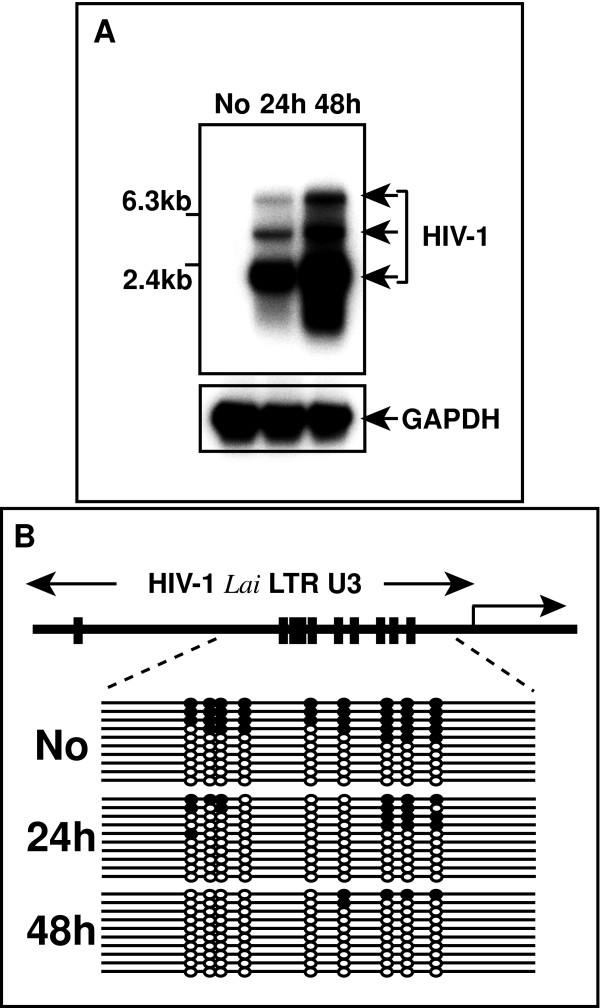
*In vitro *induction of HIV gene expression in ACH-2 cells, and demethylation of CpGs in the LTRs. **A**. Induction of HIV mRNA in ACH-2 cell line by TNF-α. Two micrograms of poly (A)+ RNA was analyzed by northern blotting. **B**. CpG demethylation after HIV induction by TNF-α. Upper panel, Schematic map of the CpG sites in the U3 region of HIV-1 IIIB LTR. The lower panel, results of bisulfite genomic sequencing. Cells are harvested at the time points indicated on the left. No, cells without TNF-α treatment. Each line represents a result of single plasmid clone. Closed and open circles indicate methylated and unmethylated CpG sites, respectively.

We then asked if TNF-α-induced HIV-1 gene expression in ACH-2 is associated with changes in CpG methylation in the LTR. TNF-α stimulation induced activation of viral gene expression after 24 hours (Fig. 2A). We used bisulfite genomic sequencing, to characterize the methylation status of nine CpG sites located in the U3 region of the HIV LTR, before and after TNF-α treatment. Bisulfite sequencing of the provirus before TNF-α treatment revealed a feature that is typical of CpG methylation (Fig. 2). The 10 plasmid clones analyzed displayed either hypermethylation or hypomethylation. Among five hypermethylated clones, three were methylated at all CpGs, and one had 7 out of 9 sites methylated. In DNA extracted from cells after 24 hours of TNF-α treatment, no alleles were completely methylated. Five of 10 sequenced plasmid clones displayed some CpG methylation, and the remaining five clones were completely unmethylated. After 48 hours of TNF-α treatment, there was a clear trend toward demethylation. Eight clones were completely unmethylated, and one had only one methylated CpG (Fig. 2B).

Taken together, the results shown in Fig. 2 can be summarized as follows: 1) at least half of the analyzed clones were unmethylated before and after TNF-α treatment, 2) the levels of CpG methylation in the remaining clones showed a clear decrease that was related to the duration of TNF-α treatment. The primers we used can amplify both 5' and 3' LTR sequences, and methylation of only half of the amplified clones suggests that methylation may affect only the 5' or the 3' LTR, as we found previously in studies of the HTLV-1 provirus [29]. On the other hand, the response to TNF-α provides evidence of cytokine receptor signal-mediated demethylation of the provirus LTR. The mechanism by which LTR is demethylated remains to be elucidated. However, our previous observation in HIV transgenic mice suggested a passive mechanism for demethylation of HIV LTR which depends on DNA replication as is supposed for demethylation of cellular genes[30].

**Figure 3:**
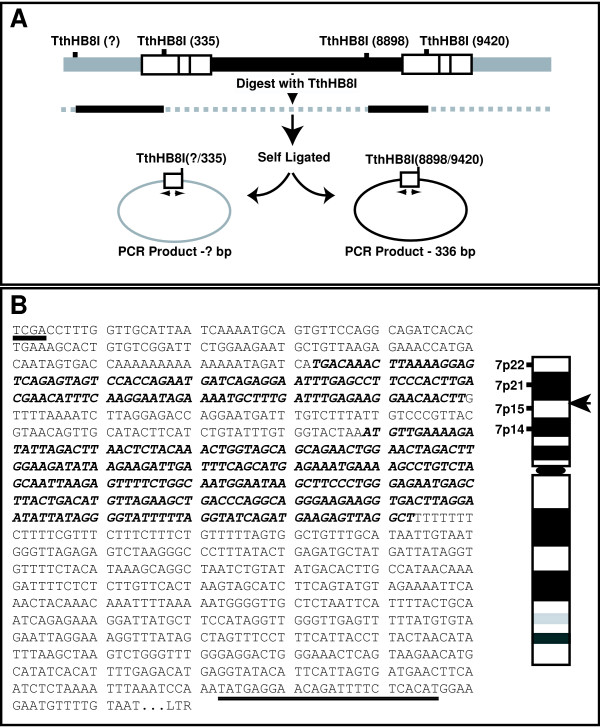
Identification of the flanking genomic sequence of the integrated HIV provirus in ACH-2 cells by the inverse PCR. **A**. A schematic chart of the inverse PCR procedure. **B**. Host genomic sequence flanking 5'-LTR. Underlined sequence indicates TthHB8I restriction enzyme site. Double underline indicates the sequence used for the sense primer in 5'-LTR specific methylation analysis. Bold italic indicates the L1 family sequences. Right panel shows a schematic presentation of the chromosomal location of the integration site of HIV-1 in ACH-2 cells determined by the NCBI human genomic blast program. Arrow indicates the region of integration.

To examine the possibility that hypermethylation is selective for the 5' LTR of the HIV provirus, we first identified the flanking genomic sequences of the integrated provirus in ACH-2 cells using the inverse polymerase chain reaction (I-PCR) [29]. Genomic DNA was digested with a restriction enzyme TthHB8I, followed by self-ligation for 12 hrs. Ligated DNA was subjected to PCR amplification using the following primers: forward primer (iHIV-2): 5'-TTCATCACGTGGCCCGAGAGCTGCATCCGGAGTAC-3' (nucleotide position: 283 to 317), reverse primer (iHIV-1): 5'-CCTTGTGTGTGGTAGATCCACAGATCAAGGATA-3' (nucleotide position: 67 to 37). Agarose gel electrophoresis of the PCR products showed two amplified bands with sizes of about 300 bp and 1,2 kbp. These bands appear to correspond to those from 5'- and 3'-flanking sequences, since ACH-2 cells contain a single copy of integrated HIV provirus per cell [31]. Both PCR products were subcloned using pGEMT-easy (Promega, Madison, Wisconsin) and the nucleotide sequences were determined. The 1.2 kbp PCR product was shown to contain the 5'-LTR sequence, and the 300 bp product that of 3'-LTR. NCBI human genomic blast program analysis of the flanking sequences showed that the sequence of the 1.2 kbp fragment corresponds to that of Chromosome 7, located at 7p15 (Fig.3B). We also identified the 5'-flanking sequences contained the L1 family repetitive sequences (Fig.3B bold italic).

**Figure 4:**
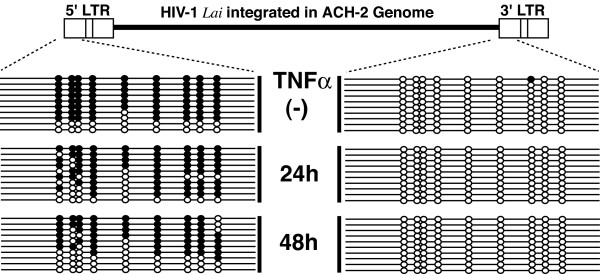
Selective analysis of CpG methylation of two LTRs. Methylation of CpG sites in each plasmid clone is displayed. Closed and open circles indicate methylated and unmethylated CpG sites, respectively. TNF-α treated cells are harvested at the time points indicated in the middle. (-), ACH-2 cells harvested before TNF-α treatment.

We used the flanking sequences to selectively characterize the CpG methylation status of the 5' LTR, preparing a forward primer located in the 5' flanking sequence. For analysis of the 3' LTR, we used a forward primer located in the nef region. The primers used are follows: 5' flanking forward primer (5'F-1) 5'-TATGAGGAATAGATTTTTTTATATG-3' (Fig.3), forward primer for 3' LTR (3'LTR-F1) 5'-TTATAAGGTAGTTGTAGATTTTAGT-3' (nucleotide position 9036 to 9060), and the reverse primer R-1 (described above). The results clearly demonstrated selective hypermethylation of the 5' LTR, with almost complete hypomethylation of the 3' LTR (Fig. 4), which was in accordance with our previous findings with integrated HTLV-1 provirus *in vivo *and *in vitro *[29].

In DNA from unstimulated ACH-2 cells, among 10 clones derived from the 5' LTR, six showed methylation of all 9 CpG sites, and two had only two unmethylated CpGs, although two clones showed complete hypomethylation of the 9 CpG sites. In contrast, all 10 clones derived from the 3' LTR were hypomethylated, with only one methylated site in one clone. The 3' LTR did not show any changes in methylation on TNF-α stimulation (Fig. 4). TNF-α stimulation resulted in increase in unmethylated CpGs in the 5' LTR. After 24 hours of TNF-α stimulation, only one clone out of 10 remained completely methylated, and most clones had one to five unmethylated sites, while one was completely unmethylated. After 48 hours of stimulation, none of the sequenced clones were completely methylated (Fig. 4). Demethylated CpGs appeared to cluster in the first 5 CpG sites (reading 5' to 3'), with higher frequency at the 5th site. Three of 10 clones from unstimulated ACH-2 cells had an unmethylated 5th CpG site, and 6 clones each had unmethylated 5th CpG site after 24 and 48 hours of stimulation. Furthermore, the number of unmethylated CpGs in the first 5 sites increased with the time of TNF-α stimulation. Before stimulation, 12 (24%) of total 50 sites were unmethylated, whereas after stimulation 22 (44%) and 26 (52%) sites were unmethylated at 24 and 48 hours respectively. On the other hand, in the cluster of 4 sites at the 3 end of the cluster, among a total of 40 sites analyzed 8 (20%) were unmethylated in unstimulated cells, and after TNF-α stimulation 6 (15%) and 17 (42.5%) sites were unmethylated at 24 and 48 hours respectively. Taken together, demethylation induced by TNF-α showed a tendency to cluster in the CpG sites located at the 5' end. Furthermore, the 5th CpG site may be a hot spot for demethylation in the TNF-α stimulated cells.

Nevertheless, our results demonstrate that in the setting of full reactivation of viral gene expression by TNF-α stimulation, the provirus LTR showed only a partial demethylation at scattered sites (Fig. 4). This observation is inconsistent with the widely accepted idea that promoter activity is regulated by the density of CpG methylation [22,32,33]. Our results may provide support for the idea that demethylation of a specific CpG site plays an important role in promoter activity of the HIV LTR, which we have previously reported using HIV transgenic mice [30]. It is also consistent with the notion that CpG methylation of specific sites plays an important role in controlling the promoter activities of EBV and imprinted genes [34-38].

Differential methylation of two LTRs located within 10 kbp of each other in the provirus genome, which we previously reported in the human retrovirus HTLV-1 [29], may suggest the presence of an unknown mechanism of methylation targeting that discriminates the 5'from the 3' LTR. The difference may depend on chemical modification of histone H3, such as lysine 9 (K9) methylation, since repression of gene expression mediated by histone H3 K9 methylation is thought to be stabilized by DNA methylation[39].

Previous studies *in vitro *suggested CpG methylation of the HIV-1 LTR as a mechanism to maintain HIV-1 latency [20-23,40,41]. However, no information is available as to the CpG methylation status of the HIV provirus in the reservoir pool *in vivo*, because extremely low copy numbers of HIV provirus make it infeasible to directly analyze CpG methylation with bisulfite genomic sequencing. In spite of the widely accepted idea that CpG methylation is involved in suppression of HIV gene expression and latency, a recent report suggested that proviral DNA methylation may not be involved in transcriptional suppression of integrated HIV provirus [42]. However, this report used an artificial system in which provirus methylation was analyzed on a defective HIV genome or a vector having only HIV LTR as the promoter. Because we lack information on the state of the latent HIV provirus *in vivo*, the notion remains to be examined.

Decipherment of the mechanisms for reactivation of latently infected HIV in the reservoir pool will provide the basis for designing treatment strategies for containment or purging of HIV. Thus our observations of 5' LTR selective methylation in ACH-2 cells, and signal-induced demethylation of HIV provirus in the transgenic mice model and latently infected cell lines [30], provide information that will be useful in future investigations.

## Abbreviations

HIV: human immunodeficiency virus

LTR: long terminal repeat

HAART: highly active anti-retrovirus therapy

I-PCR: inverse polymerase chain reaction

## Competing interests

The author(s) declare that they have no competing interests.

## Authors' contributions

T.I. carried out experiments and contributed to data analysis, interpretation, and preparation of figures and drafted the manuscript. A.H. and T.K. carried out part of the experiments including CpG methylation analysis and participated in data analysis and interpretation. T. W. conceived the study, and participated in its design and coordination, and drafted the manuscript.
